# An atomistic workflow for determining acid–base prototropic tautomer microstate populations

**DOI:** 10.1039/d6ra08700k

**Published:** 2026-11-03

**Authors:** Jonathan W. Zheng, Esther Heid

**Affiliations:** a Institute of Materials Chemistry, TU Wien A-1060 Vienna Austria esther.heid@tuwien.ac.at

## Abstract

We present a new cheminformatics package, Protomer Enumerator & Automatic Calculator for Energies (PEACE), which lists the prototropic forms of a given acid or base, and predicts which of these microstates are likely to be present in solution. The workflow enumerates prototropic tautomers, and orchestrates inexpensive semi-empirical quantum chemical simulations to evaluate their single-point energies, solvation free energies, entropic contributions, and ultimately their solution-phase energies. The relative population of each tautomer is then calculated. PEACE includes automatic error checks, tautomer screening, and conformer generation to improve speed and accuracy. It can be run in several solvent systems and on charged solutes. Herein, we demonstrate the workflow's efficacy on numerous practical benchmarks including on druglike molecules. The workflow is freely available for use through the command line with minimal package dependencies.

## Abstract 2

PEACE is an atomistic workflow for enumerating prototropic tautomers, usable for identifying ionization centers and quantifying tautomer equilibria.

## Graphical abstract

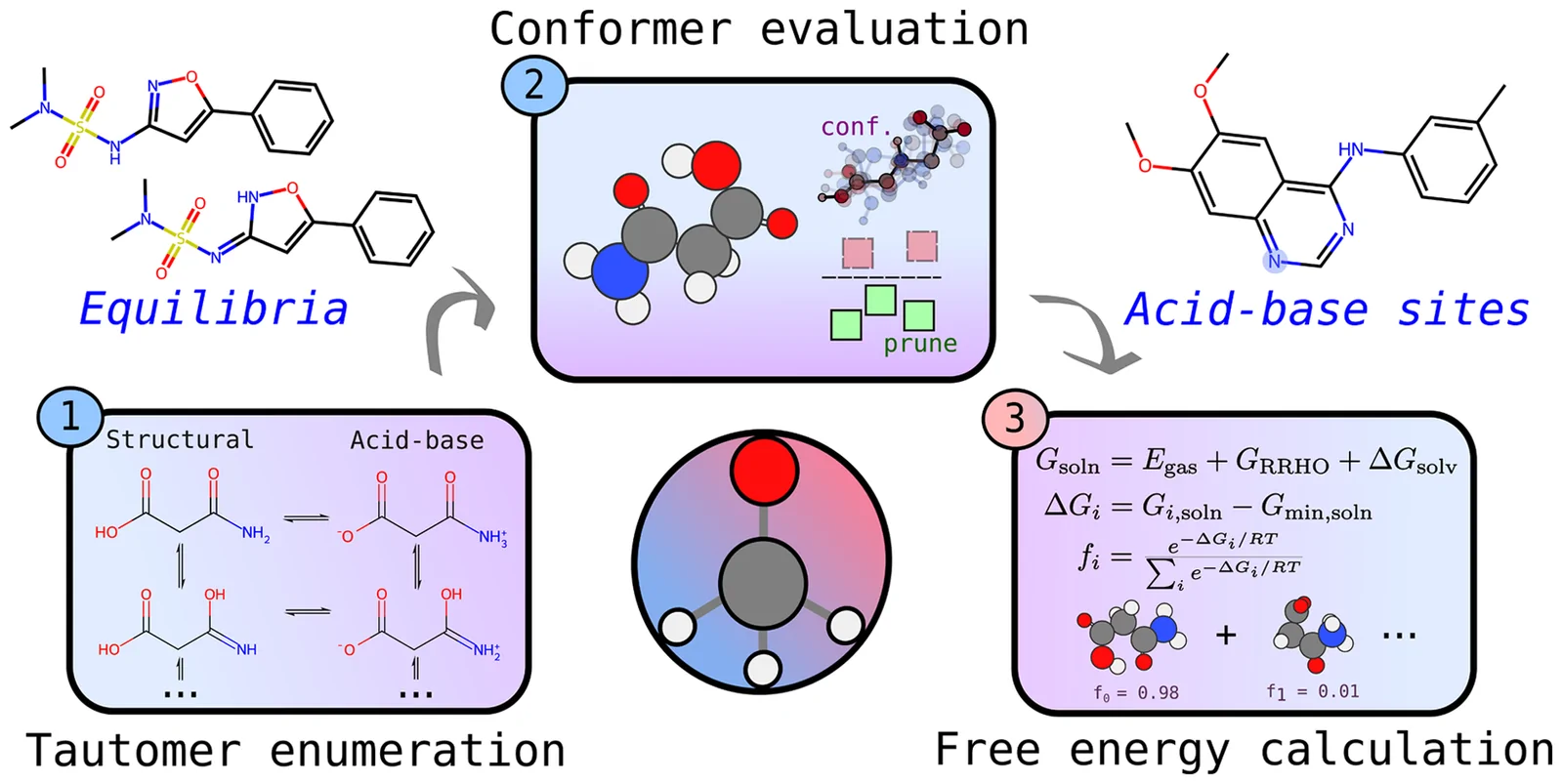

## 1 Introduction

Protonation and deprotonation are widespread chemical reaction processes. However, workflows for determining prototropic tautomers (here, protomers) arising from such (de)protonation reactions are not standardized, and often require task-specific workflows which can be slow to use or difficult to adapt.^1–4^ This can frustrate machine learning and atomistic simulations by complicating which tautomer(s) to include.

To determine relevant species arising from acid–base phenomena, one can estimate the relative solution-phase energies of species, usually through the acid dissociation constant, or p*K*_a_. In recent years, quantum chemical simulations^5,6^ have been used, and newly-digitized datasets^7–9^ have been made available for determination of p*K*_a_. However, the p*K*_a_ data (which are overwhelmingly macroscopic values, corresponding to ensembles of chemical species) are difficult to compare with values from simulations, which typically examine only one tautomeric form at a time. Furthermore, machine learning models have often been trained with only one “representative” form of an acid. This has led to some issues with modeling amphiprotic compounds, as sometimes a minor form is chosen, or in certain cases multiple tautomeric forms are important rather than just one.^10,11^

Bridging macroscopic experimental measurements with microscopic calculations requires use of workflows that account for protomers, but such open-source methods are currently few in number. One included in CREST^12–14^ systematically (de)protonates all suitable hydrogen atoms and evaluates the tautomer rankings using GFN2-xTB.^15^ Because the selection of (de)protonation sites is exhaustive, spurious tautomers are often selected, and the accuracy of the quantum chemical methods is not often sufficient to determine which sites are majorly important. Another method was developed by Luo *et al.* for p*K*_a_ prediction,^3^ and slightly modified in 2025 by Wagen of Rowan Scientific.^16^ This approach enumerates protomers based on a list of manually compiled acid–base substructures, and then computes relevant tautomers' energies using machine-learned free energy models. Though this approach represents a promising advancement, it was noted by both Luo *et al.*^3^ and Wagen that the approach struggles with accurate determination of tautomer energies. These methods are also parameterized only for aqueous tautomers.

Other tools for tautomer enumeration are available in some proprietary toolkits. Most use 2D structure-based methods, usually for tautomer enumeration.^17–19^ These methods are fast, but are limited to aqueous systems and typically do not estimate microstate populations – and, again, are not open source. Other proprietary methods include physics-based workflows,^20^ but there is not yet an open-source workflow that works for prototropic tautomers. Some cheminformatics tools, such as Dimorphite-DL,^1^ enumerate relevant tautomers at a given pH based on p*K*_a_ heuristics, but do not estimate microstate populations (and, again, are limited to predictions in water).

In this work, we introduce PEACE, a physics- and graph-based workflow for determining energetically-relevant prototropic tautomers. The model leverages state-of-the-art semi-empirical quantum chemical methods to achieve a balance of speed and accuracy for assessing tautomer energies. Such model outputs can be used for screening purposes, data pre-processing for downstream modeling, or direct estimation of solution-phase thermodynamic properties. The workflow is depicted in Fig. 1.

**Fig. 1:**
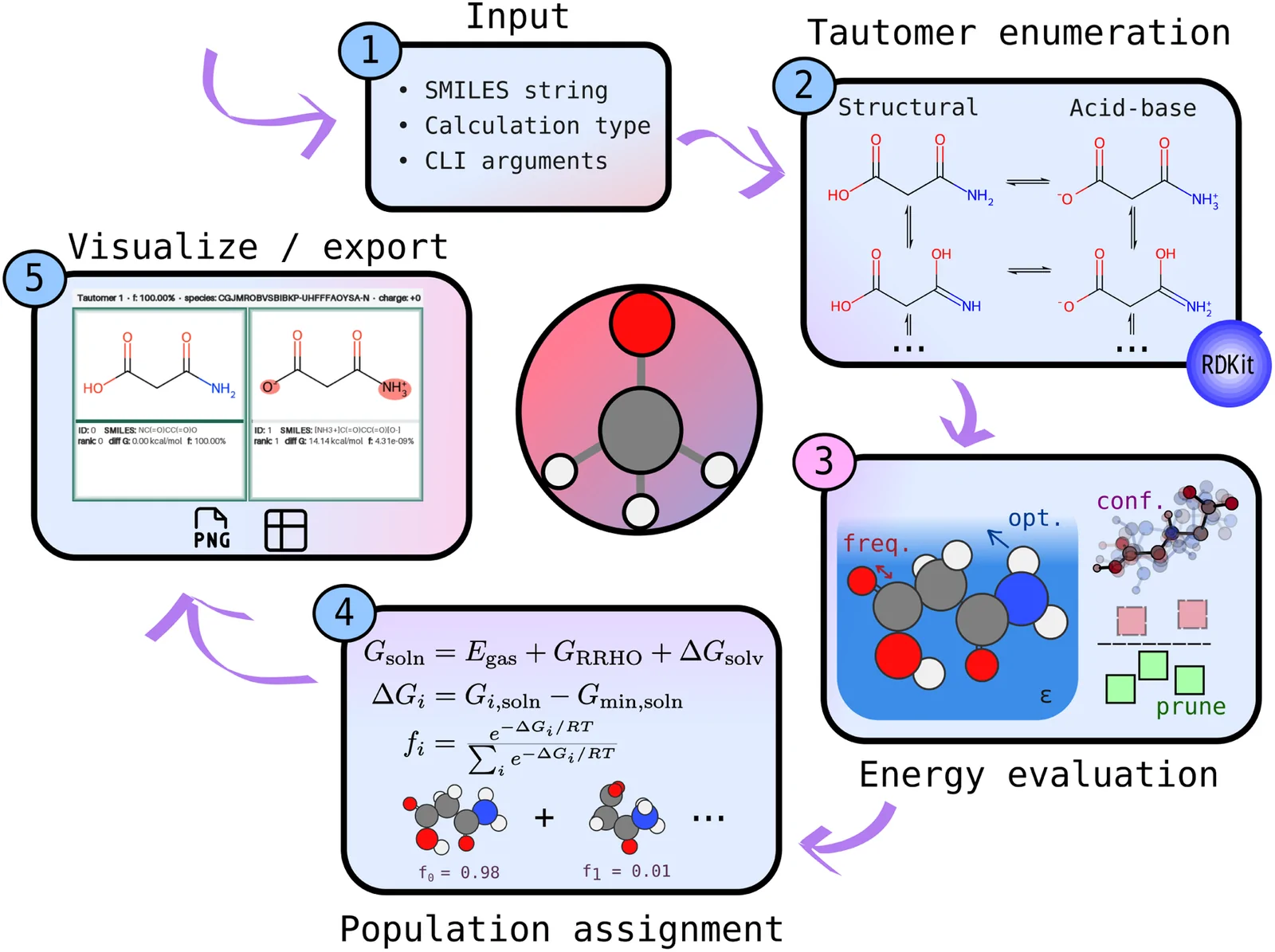
PEACE workflow. Based on an input tautomer, a list of protomers is generated, energetically evaluated, Boltzmann-weighted, and finally exported.

## 2 Methods

### 2.1 Workflow overview

PEACE starts by analyzing the input tautomer, provided by the user as a SMILES string. It is instantiated as a Mol object in the RDKit,^21^ and calls the RDKit's TautomerEnumerator to generate structural tautomers, *i.e.* a π-bond shift accompanying an H-atom shift, such as keto–enol and enamine–imine tautomerization.

To identify zwitterions, PEACE lists out each pair of (de)protonated acid-base sites for each structural tautomer. Similar to previous approaches,^3,22^ we manually enumerated a list of relevant SMARTS substructures representing (de)protonation. In order to enumerate only common, physically relevant sites, we kept the list small (currently only 11 sites). We labeled these sites as “strong” or “weak”; PEACE by default starts with “strong” and falls back to “weak” only if strong sites are not found. To validate that these sites were comprehensive, we checked PEACE against all non-salt compounds in the aqueous IUPAC Digitized p*K*_a_ Database. We verified that PEACE could find at least one protomer for the vast majority of dissociations in the dataset. For instance, for the p*K*_a4_ of EDTA, corresponding to the deprotonations of the thrice-deprotonated (charge −3) to fourfold-deprotonated (charge −4) states, we ensured that PEACE could identify valid species for both its −3 and −4 charge states, starting from a neutral tautomer. This procedure even helped us identify a few errors in the source data, which were corrected. We note a few cases that PEACE cannot yet handle: Lewis acids/bases, π-bonding bases, ring closures, very weak bases like acetophenone and benzoic acid derivatives, and some special carbon acids like cyclopentadiene. Future versions of PEACE may account for these.

For enumerating tautomers at non-zero charge, PEACE takes all enumerated tautomers from the previous charge state and exhaustively (de)protonates at acid–base sites. Unlike for zwitterion detection, PEACE also considers “very weak” sites if it cannot find any “strong” or “weak” ones. PEACE also adds any structural tautomers from TautomerEnumerator and zwitterions that were not available using tautomers at the previous charge state. This entire process is repeated until the desired charge state is reached.

At this point, the user can visualize all enumerated protomers. However, often the majority will be unimportant. We therefore include a screening approach based on solution free energies (*G*_soln_), computed *via*:*G*_soln_ = *E*_gas_ + *G*_RRHO_ + Δ*G*_solv_where *E*_gas_ is the electronic free energy, *G*_RRHO_ is the thermal contribution to free energy, and Δ*G*_solv_ is the solvation free energy.^23,24^ For the purpose of this communication, all results were made using the g-xTB^25^ for single-point energies, GFN2-xTB for frequencies,^15^ GFN2-xTB/ALPB^26,27^ for optimization, and CPCM-X^28^ for solvation free energies. These calculators are modular; they may be changed in future versions, and users may also use QM methods available in ORCA 6. The calculators used herein were chosen on the basis of balancing speed and accuracy. g-xTB and GFN2-xTB are semi-empirical quantum mechanical methods, which are orders of magnitude faster than *ab initio* quantum mechanical methods at a slight cost in accuracy. g-xTB replicates ωB97M-V^29,30^/def2-TZVPPD^31–33^ energies. Meanwhile, GFN2-xTB is less accurate, but is used herein for frequency calculations (though the user can specify to use g-xTB instead), because it is faster than g-xTB with only slightly worse accuracy. It is also used for the initial solvation optimization, because (at present) implicit solvation models are not fully implemented in g-xTB. ALPB is fast implicit solvation model used for optimization; CPCM-X is more accurate but slower, so it is used only for the single-point solvation energy calculation.

PEACE first performs a rough energy screening. Each protomer is embedded with one KDG^34^ conformer. The solution free energies are calculated as above, and used to screen in the most important protomers under a user-defined energy threshold. Surviving protomers undergo a conformer generation step: conformers are generated using KDG, and then relaxed in MMFF94.^35–39^ The lowest-energy conformers from the pool are then optimized with GFN2-xTB/ALPB. The solvation free energy is calculated on each conformer. By default, PEACE then further relaxes the structure in the gas phase using g-xTB, which if successful is then used (otherwise falling back on the ALPB geometry) to calculate the gas-phase single-point energy and thermal contributions. The solution free energy of all conformers is finally Boltzmann weighted to give *G*_soln_ for the protomer. The *G*_soln_ of all protomers is again Boltzmann weighted to yield population microstate fractions, as follows:
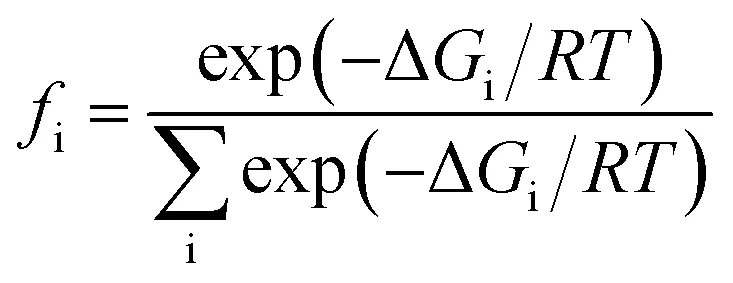
where *f*_i_ is the microstate population fraction, Δ*G*_i_ is the protomer solution free energy relative to the most stable protomer, *R* is the gas constant and *T* is temperature.

Quality assurance checks are performed at each step. Whenever a structure is optimized, its graph connectivity is checked against the input Mol, and resonance structures are detected and removed. Conformer pools, both before and after optimization, are pruned using PRISM Pruner.^40^ Conformer pools before optimization are also pruned based on energies, to exclude physically implausible conformers. xTB calculation errors are automatically identified and, if possible, calculations are re-run with higher electronic temperatures to improve SCF convergence, or with looser optimization thresholds (and logged to inform the user). All steps are rigorously logged and reported to the user, including quantum mechanical (QM) job failures. QM calculations for zwitterions containing NH_3_^+^ motifs are also constrained to avoid hydrogen abstraction in gas-phase optimization (if performed). Nearly all steps and parameters of the workflow are customizable through CLI arguments, such as the maximum number of conformers, temperature (affecting the thermal contributions and Boltzmann weighting, but not yet the solvation energy temperature dependence), screening energy thresholds, maximum number of conformers to include, and others.

### 2.2 Benchmark data preprocessing

We validated PEACE against a variety of benchmarks, running the full solution free energy-based microstate workflow at room temperature:

(1) SAMPL6: three NMR-resolved and seven inferred protonation microstates for kinase inhibitor-like fragments, at charge states between −1 and 2.^41,42^ We obtained the structures from SMILES strings in the SAMPL6 GitHub repo with commit hash dfb327b and verified them against their published structures. We used PEACE to predict the charged microstates of the kinase inhibitor-like fragments.

(2) SAMPL7: numerous microstates between −1 and 1 formal charge, enumerated by the RDKit and validated as the union of three proprietary cheminformatics software outputs. Free energy differences between microstates were computed by SAMPL7 challenge entrants, and provided online.^43^ SMILES strings were obtained from the SAMPL7 GitHub repository with commit hash 114e35a. We verified that the strings matched the structures visualized in the repository. We only chose compounds where multiple non-stereoisomeric microstates were identified for at least one charge state.

We ran PEACE on six different SAMPL7 compounds across −1, 0, and 1 charge states, depending on the compound. This yielded ten separate test cases, for which we compared the PEACE-predicted tautomer energies to the average of those provided by participants in SAMPL7. We note that SAMPL7 did not publish any experimental values for microstate energies or populations, so the participant averages act as a best guess for the correct relative tautomer energies.

(3) Amino acids: 20 canonical amino acids in water (the zwitterion form dominates in all cases). We visually double-checked that the l-amino acid SMILES strings matched their well-known structures. Also, we extracted data from the literature for 18 canonical and non-canonical amino acids in dimethyl sulfoxide, with experimentally measured *f*_zwit_ between 0.04 and 0.98.^44,45^

(4) Realistic molecules: 40 molecules, including drug and druglike molecules,^46–50^ neurotransmitters,^51,52^ vitamins,^49^ and small molecules,^53–55^ with ratios of their zwitterionic and uncharged microstate populations in water. We compiled these data from numerous sources in the literature, in some cases interconverting between *K*_zwit_ (tautomerization equilibrium constant) and *f*_zwit_ (fractional population of zwitterions). As temperature was almost always unspecified, we assumed a temperature of 298 K.

(5) Gimadiev tautomer set: a set of 785 tautomer transformations, mostly uncharged (*e.g.* keto–enol, amino–imino), was originally compiled by Gimadiev *et al.*^56^ from the Palm handbook.^57^ From this, we downsampled to pure solvents that can be modeled with PEACE, resulting in 228 entries. Among these, 36 of the tautomerisms could not be modeled (34 ring closures and 2 other prototropic tautomers not captured by PEACE). This resulted in 192 tautomerisms.

In an initial run of PEACE, we found that PEACE-predicted log_10_ *K*_zwit_ had similar magnitudes to the experimental data, but in opposite directions of equilibria. This led to huge errors, up to 26 log_10_ units in some cases. Upon further inspection, we determined that the tabulated constants defied chemical intuition. For instance, aminopyridine compounds' pyridinoid tautomers were orders of magnitude less favored than the pyridonoid forms, which lack the resonance stability of the other isomers. We checked the original data sources, and identified that the source of the errors came from the Palm handbook flipping the tautomerization equilibria. We found, in total, 12 errors, 11 of which we could correct by adding a negative sign, and 1 of which we removed. The final dataset analyzed here thereby includes 191 data, with 11 of them corrected; 165 are uncharged and 26 are zwitterionic. The downsampled data include temperatures close to 298 K, and cover the solvents acetonitrile, benzene, DMF, DMSO, methanol, nitromethane, THF, toluene, and water.

Finally, in addition, we extracted a test set from the Gimadiev study, downsampling from 26 datapoints to 14, correcting 1 equilibrium direction as before.

All benchmark data are available in tabulated.csv form, along with benchmark results, in the GitHub repository https://github.com/jonwzheng/PEACE_experiments.

## 3 Results

### 3.1 SAMPL6

For all eight fragments, PEACE obtained the correct uncharged tautomer (Fig. 2). In all but one of the nonzero charge test cases, PEACE properly identified the dominant microstates (*i.e.*, the protonation or deprotonation sites) in agreement with the experimental data. For the single failure case (SM14), PEACE identified the correct sites, but the incorrect order of protonation (*i.e.* incorrect +1 microstate).

**Fig. 2:**
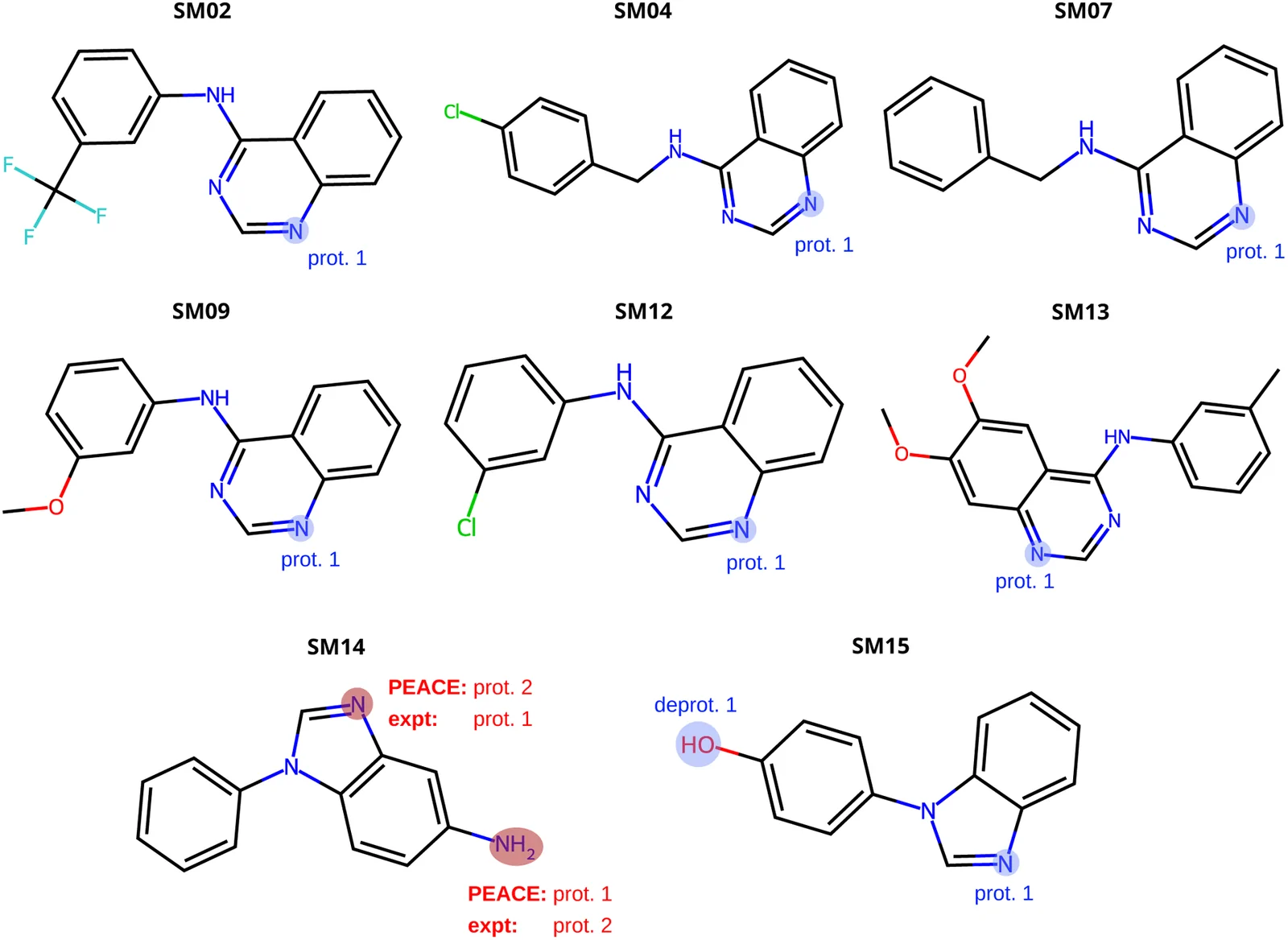
Species in SAMPL6. acid–base sites are colored in blue if PEACE tautomers agree with experiment, otherwise in red. “Deprot” and “prot” here refer to (de)protonation at the respective site.

### 3.2 SAMPL7

In all cases, PEACE identified all of the SAMPL7-enumerated important microstates, correctly ranking them within the top three tautomers. Fig. 3 shows one such example. Furthermore, in all but two cases, the single most dominant protomer predicted by PEACE comports with the lowest-energy microstate predicted in SAMPL7. In those two errant cases, the SAMPL7 energy prediction spreads were very large (standard deviations of 20.5 and 25.0 kcal mol^−1^), making direct comparison with those essentially meaningless. These results show that PEACE qualitatively obtains, within just a few minutes, a realistic tautomer pool.

**Fig. 3:**
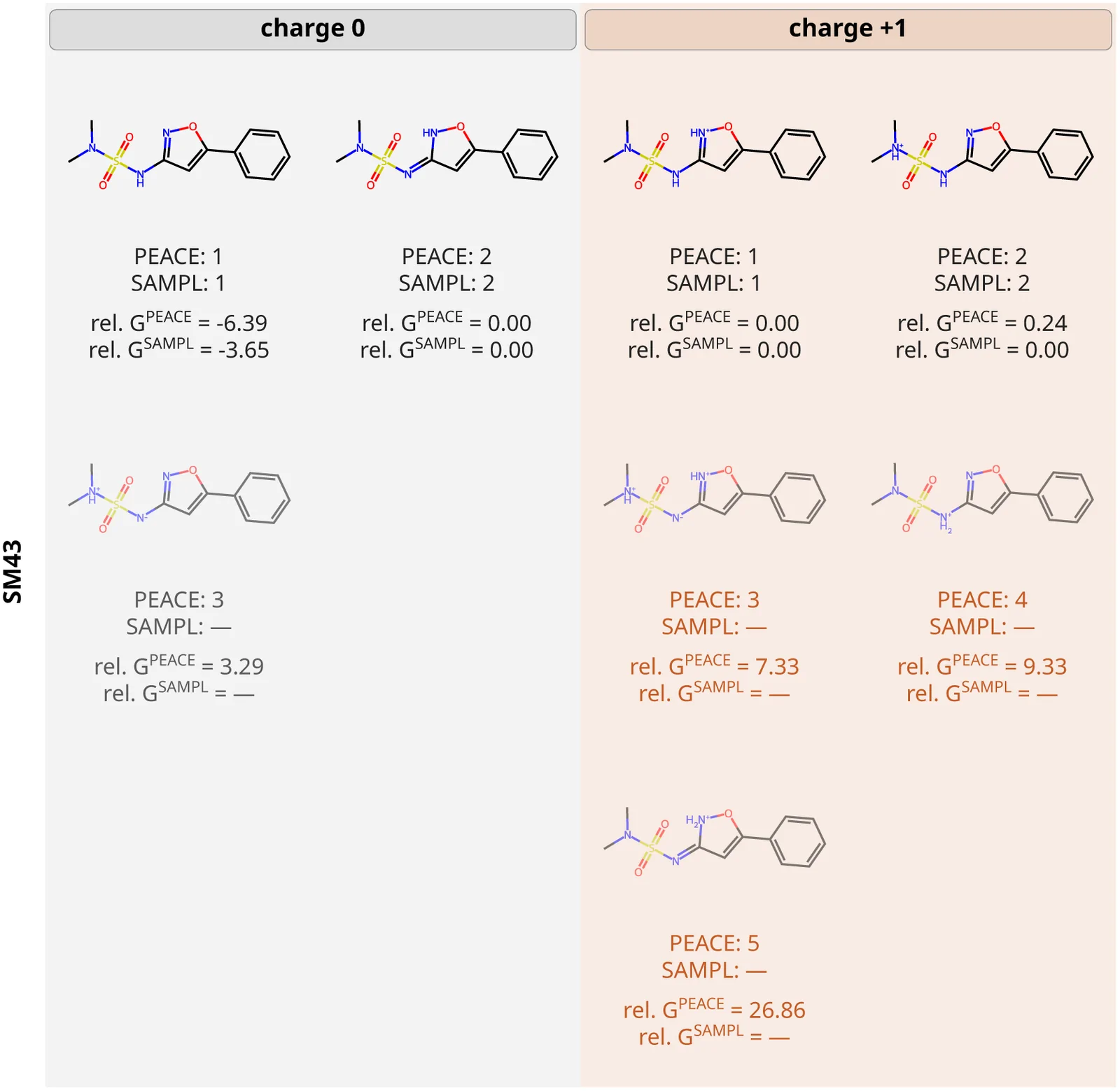
Example of tautomers enumerated by PEACE for species SM43. Tautomers found by both PEACE and SAMPL7 are shown as opaque, whereas those only in PEACE are shown with transparency and off-black text. Protomer rankings (*e.g.* lowest solution free energy, relative to the SAMPL7 “canonical” tautomer) are shown below each structure. In this case, the SAMPL7 energy predictions and rankings comport with the PEACE microstate predictions.

### 3.3 Amino acids

For the 20 canonical amino acids in water, PEACE predicts that the *f*_zwit_ for all of them exceeds 0.999, indicating zwitterion dominance. This comports with experiment.^58^

Among the 18 amino acids in dimethyl sulfoxide, PEACE predicts the correct major form in 12 cases. In the six other cases, PEACE strongly underpredicts the zwitterion population. The issue is most pronounced for species with *f*_zwit_ in intermediate ranges, *e.g.* between 0.10 and 0.90, as minor errors in free energies can cause large fluctuations in *f*_zwit_ in this region. In contrast, PEACE correctly predicts the major species for eight of the nine cases where zwitterions strongly dominate (*f*_zwit_ > 0.90).

### 3.4 Realistic molecules

Fig. 4 shows the PEACE-predicted *vs.* experimentally-measured log_10_ *K*_zwit_ values (where 
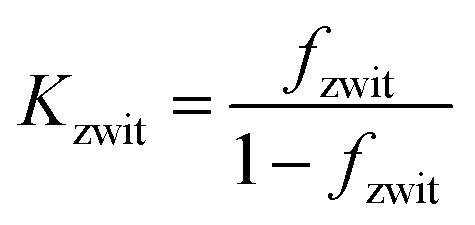
, representing the numerical ratio of zwitterionic and uncharged microstates). All such molecules are at charge state 0. The results show that generally, PEACE predicts correctly whether the zwitterion or neutral species dominates. However, the quantitative agreement is poor, often over- or under-estimating ratios by several orders of magnitudes in error. In the overprediction regime, this is not necessarily an issue (the difference, for instance, between 99% and 99.99% zwitterion is not usually important). But in the intermediate regime, *e.g.* between 10–90%, PEACE often fails to capture the major species, just as for the amino acids DMSO test case.

**Fig. 4:**
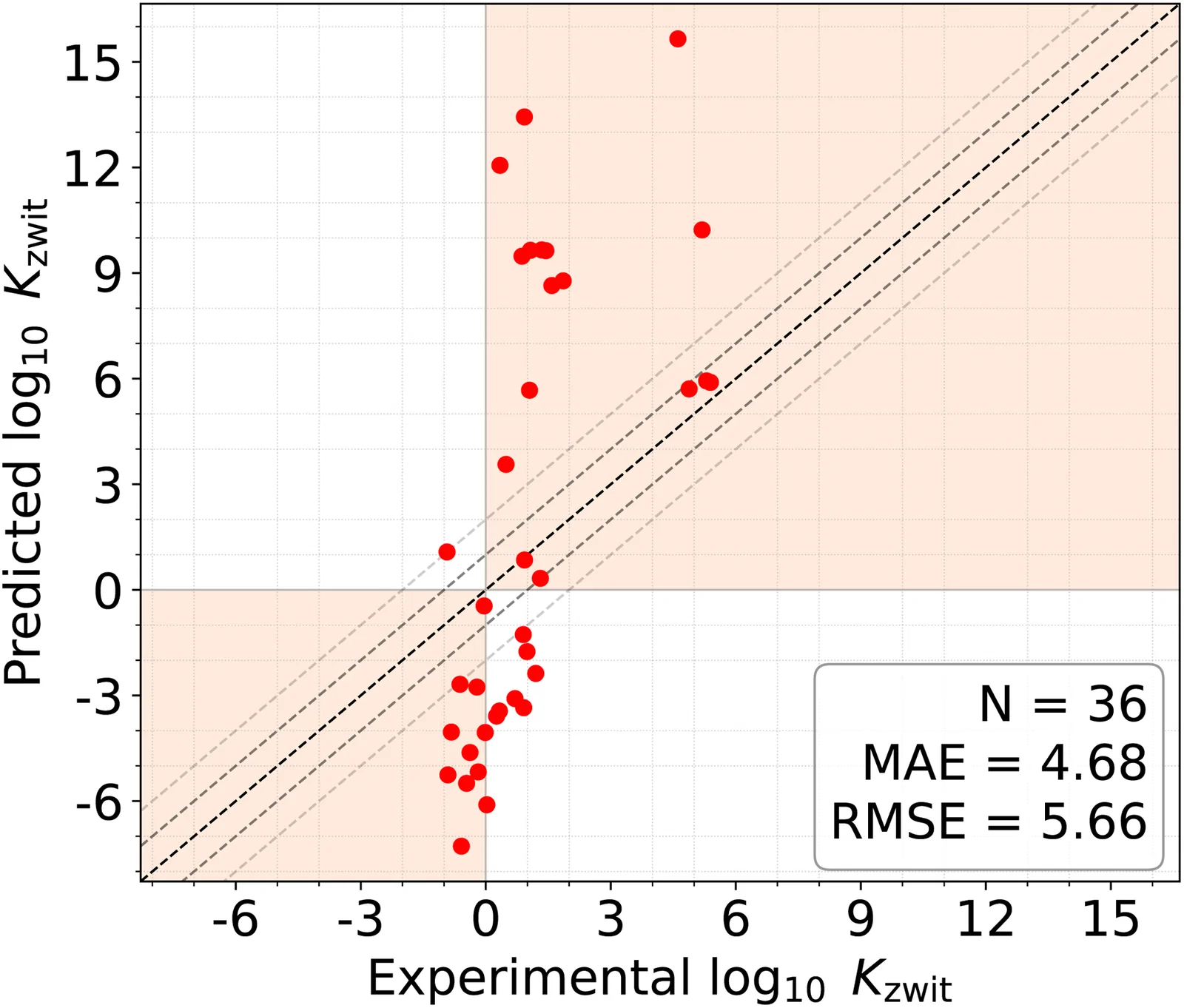
Benchmark of realistic molecules, where *K*_zwit_ is the ratio between zwitterionic and uncharged microstate concentrations. Orange regions indicate areas where both the experimental and predicted *K*_zwit_ agree on the major form.

### 3.5 Gimadiev tautomer set

The performance of PEACE on the main Gimadiev tautomerization set is shown in Fig. 5, showing generally good qualitative agreement. Across the 191 tested compounds, PEACE achieved an MAE of 2.03 and RMSE of 2.93.

**Fig. 5:**
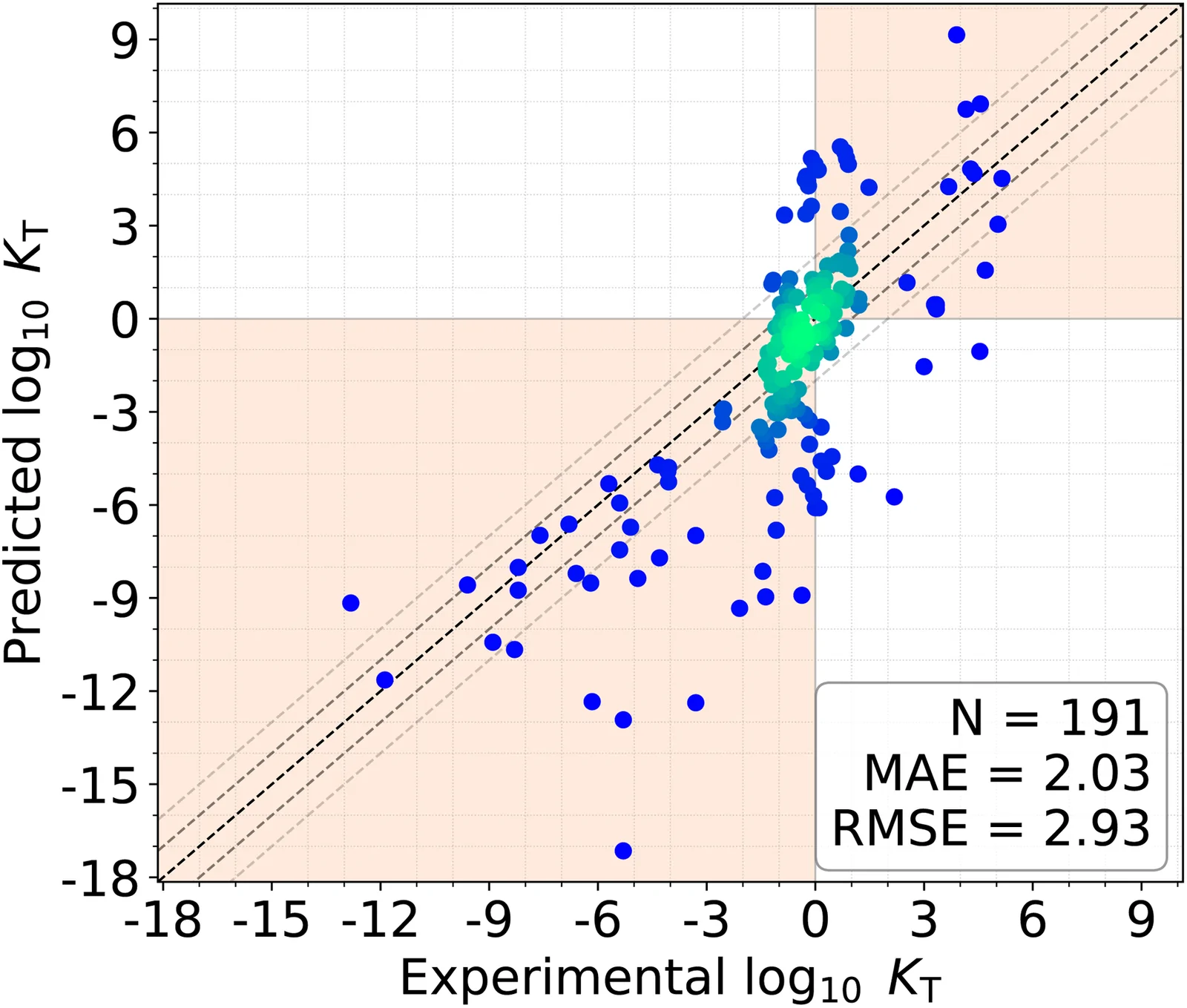
Benchmark of Gimadiev tautomerizations, where *K*_T_ is the tautomerization constant. Orange regions indicate areas where both the experimental and predicted *K*_T_ agree on the major form. Lighter points indicate higher density of points.

The work by Gimadiev *et al.* also reported RMSEs of 0.66 and 1.63 against two test splits with a data-driven method, and RMSEs of 1.1 and 3.00 using DFT. However, data for tautomerization constants were only provided for the second (more challenging) test set, which we had to downsample.^56^ Hence, we do not have a directly analogous comparison to the Gimadiev results. The PEACE benchmarks against the downsampled set resulted in an MAE of 2.53 and RMSE of 3.35. The qualitative performance of our approach was good, with only 3 datapoints disagreeing on the major form (parity plot shown in the SI).

Data-driven approaches appear to provide better performance than PEACE for test examples similar to the training data. However, the physics-based approach of PEACE had some advantages: it led us to identify large errors (up to 26 log_10_ units!) in the source data during data curation, which would have been difficult with only a data-driven method. PEACE also has the benefit of expressing tautomerization ratios among arbitrarily many isomers, and can be used for any charge state. Finally, although the numerical errors of PEACE are high, they are similar to DFT-level errors at only a fraction of the computational cost; all our experiments were run on a single laptop CPU within minutes.

## 4 Conclusion

Considering all benchmarks, PEACE provides good qualitative microstate determination, and in most cases identifies the important protomers. However, more work must be done to improve its quantitative treatment of microstate populations, owing to the sensitivity of the Boltzmann populations to free energies in the intermediate regions.

PEACE is suitable for protomer enumeration of acids and bases, and includes several tools to allow estimation of microstate populations, which have been benchmarked and demonstrated to yield qualitatively accurate performance across several challenge benchmarks, though quantitative error for individual tautomer pairs remains high. Its quantitative accuracy is enough to generally determine correct microstates and relative (de)protonation trends, but requires further improvement to more accurately distinguish among similar-energy tautomers.

PEACE provides a means of bridging atomistic simulations with experimental measurements for ionizable compounds. The accuracy and speed of the method can be tailored by adjusting the simulation settings, *e.g.* the number of protomers to pass through initial screening and the number of conformers per tautomer to consider.

Future work will focus on understanding the primary sources of error (*e.g.* gas-phase electronic energies or solvation free energies) as compared to high-level quantum chemistry calculations. Alternative solvation schemes (*e.g.* cluster continuum or explicit solvation) should be considered. We also observed that significant errors can occur if key conformers are not found during conformer enumeration; further work should be done to improve rapid solution-specific conformer generation workflows. Finally, a machine learning predictor to replace the individual microstate simulations would further accelerate this workflow, enabling its use for high-throughput screening and on-the-fly modeling.

## Conflicts of interest

There are no conflicts to declare.

## Supplementary Material

RA-OLF-D6RA08700K-s001

## Data Availability

The PEACE source code is available at https://github.com/jonwzheng/PEACE/issue/34. The code for the experiments conducted in this work are available at https://github.com/jonwzheng/PEACE_experiments. Supplementary information (SI): additional benchmarking details and corrected experimental data for tautomer ratios. See DOI: https://doi.org/10.1039/d6ra08700k.
