# Supplementary material for: An atomistic workflow for determining acid–base prototropic tautomer microstate populations
Source: RSC Adv. 2026 Nov 3. Online ahead of print. doi: 10.1039/d6ra08700k (PMC13730536; doi:10.1039/d6ra08700k)
Supplement: Supplementary file 1 [file RA-OLF-D6RA08700K-s001.pdf]

Supplementary Information (SI) for RSC Advances.
This journal is © The Royal Society of Chemistry

Supporting Information: Supporting Information for: An Atomistic Workflow for Determining Acid-Base Prototropic Tautomer Microstate Populations

Jonathan W. Zheng and Esther Heid*

Institute of Materials Chemistry, TU Wien, A-1060 Vienna, Austria

E-mail: esther.heid@tuwien.ac.at

Contents

1 Benchmarks **S1**

1.1 Amino acids S2

1.2 SAMPL6 . . . . S3

1.3 SAMPL7 . . . . S3

1.4 Realistic molecules S10

2 Benchmarking parameters **S11**

3 Corrected Gimadiev values **S11**

1 Benchmarks

All benchmarks are available at: github.com/jonwzheng/PEACE_experiments/.

1.1 Amino acids

The 20 canonical amino acids (with L-stereochemistry) structures were obtained from Wikipedia and manually checked for correctness. The benchmarks on this set are shown in Figure S1.

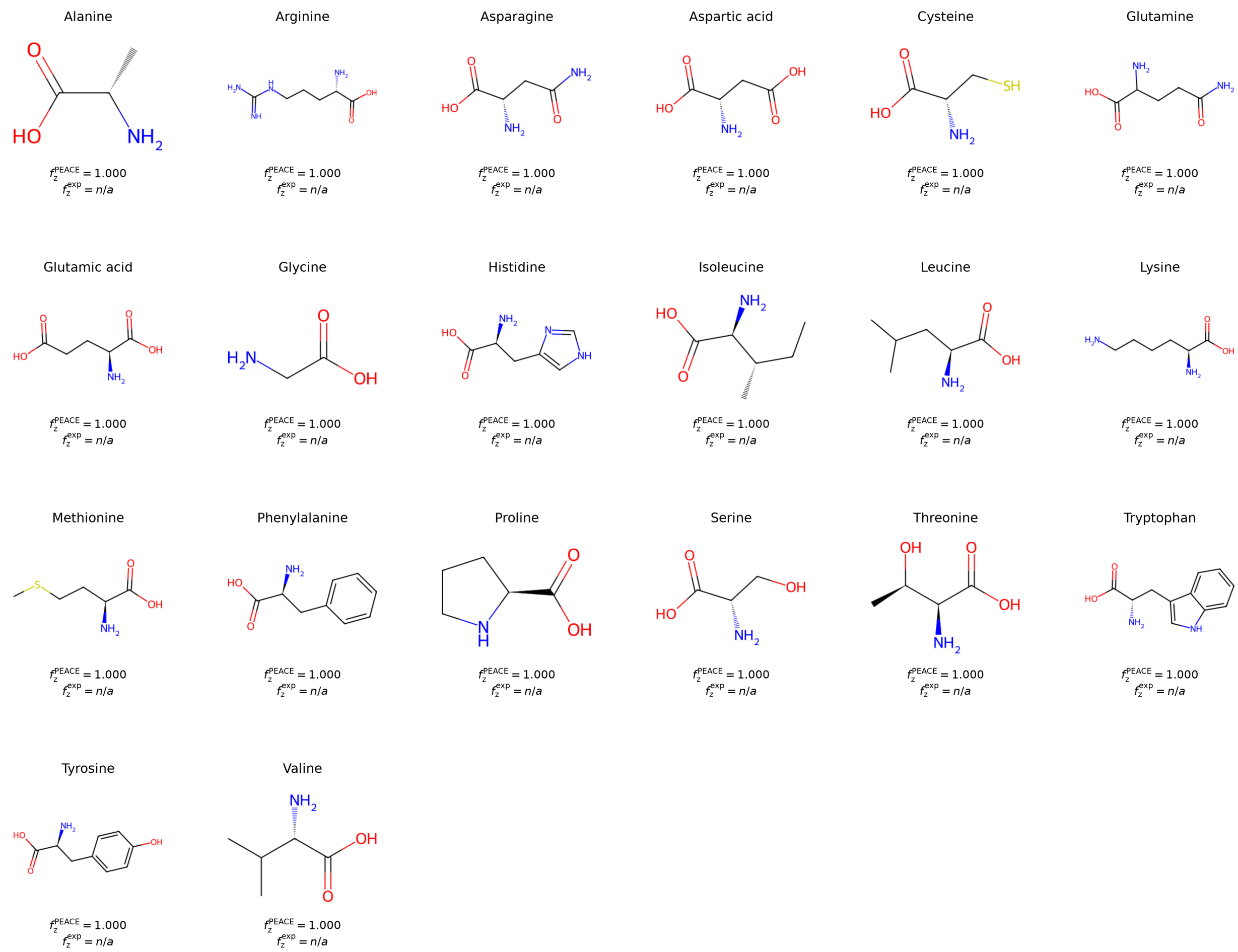

Figure S1: Predicted microstates and populations for the aqueous amino acids set.

The non-aqueous amino acids were obtained from various sources in academic literature. The original source works can be found from the benchmarks GitHub repository provided above. Benchmarks are shown in Figure S2.

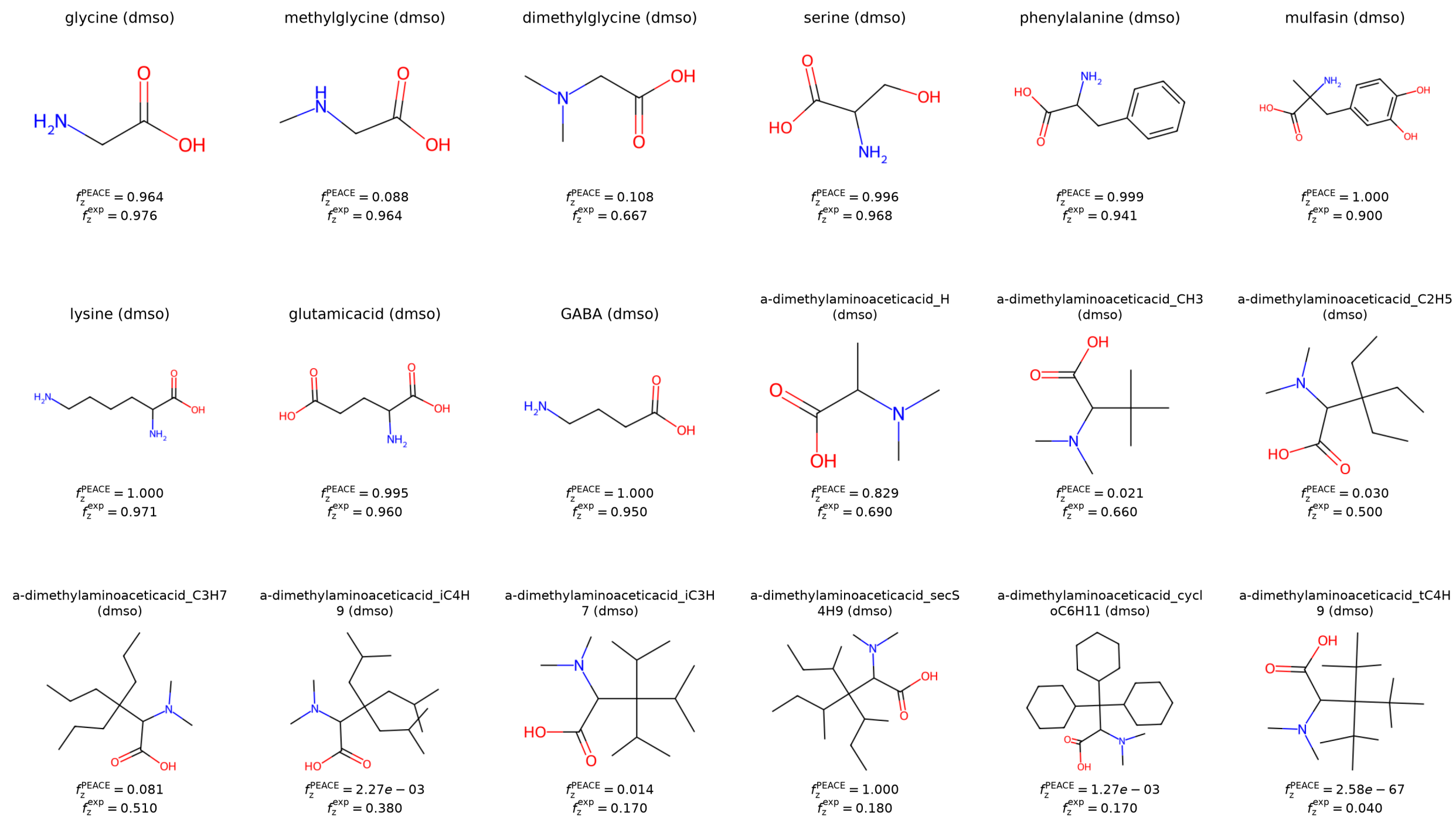

Figure S2: Predicted microstates and populations for the non-aqueous amino acids set.

1.2 SAMPL6

SAMPL6 molecules were obtained from the SAMPL6 GitHub (commit hash c661d39). Results are shown in the main text.

1.3 SAMPL7

All energies shown herein are relative to "canonical" tautomers. For charge=0, this means state `_micro001`; for other charge states, this is simply the lowest energy tautomer that charge state. We used the SAMPL7 "average" predictions as reported in 'numbers.csv'. Microstates and their energies are shown below in Figures S3, S4, S5, S6, S7, S8.

SAMPL7 molecules were obtained from the SAMPL7 GitHub (commit hash 56c855e). We downsampled to only those with at least two microstates for at least one charge state. Note, we removed stereochemistry for `SM26_micro004`, to match the canonical structure shown elsewhere in SAMPL7, and so it could properly match PEACE for benchmarking.

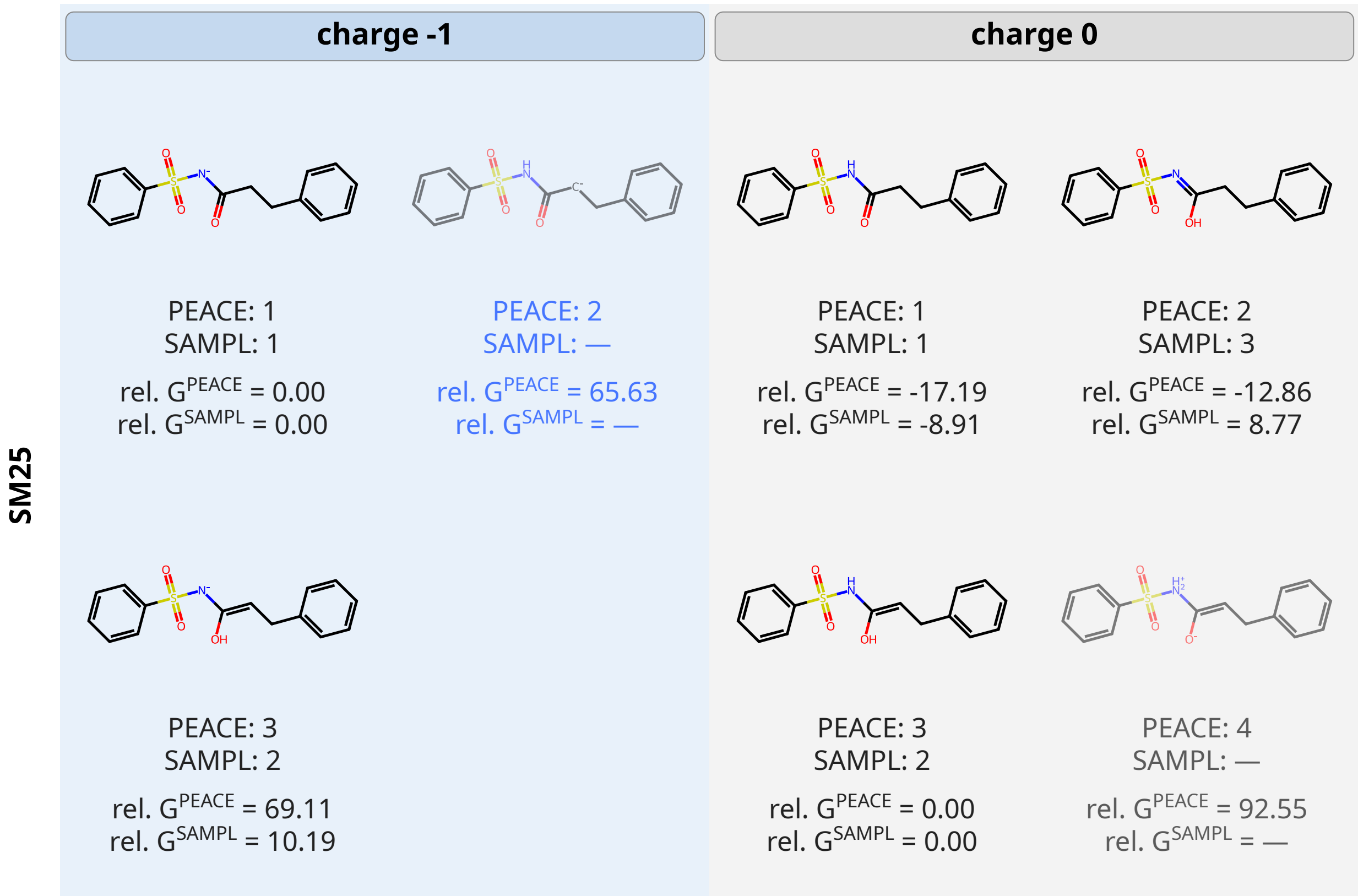

Figure S3: Predicted microstates and energies for species SM25.

SM26

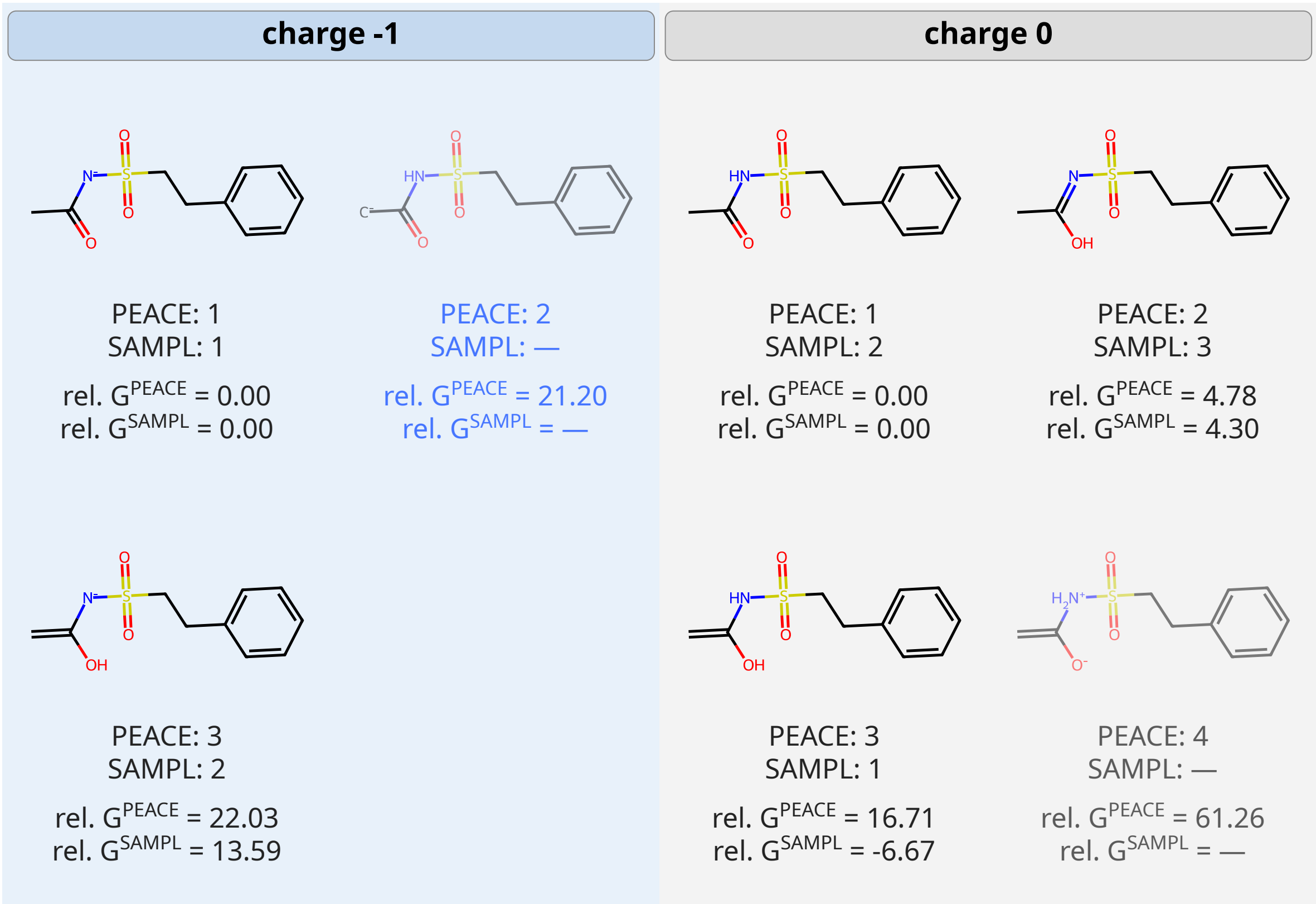

Figure S4: Predicted microstates and energies for species SM26.

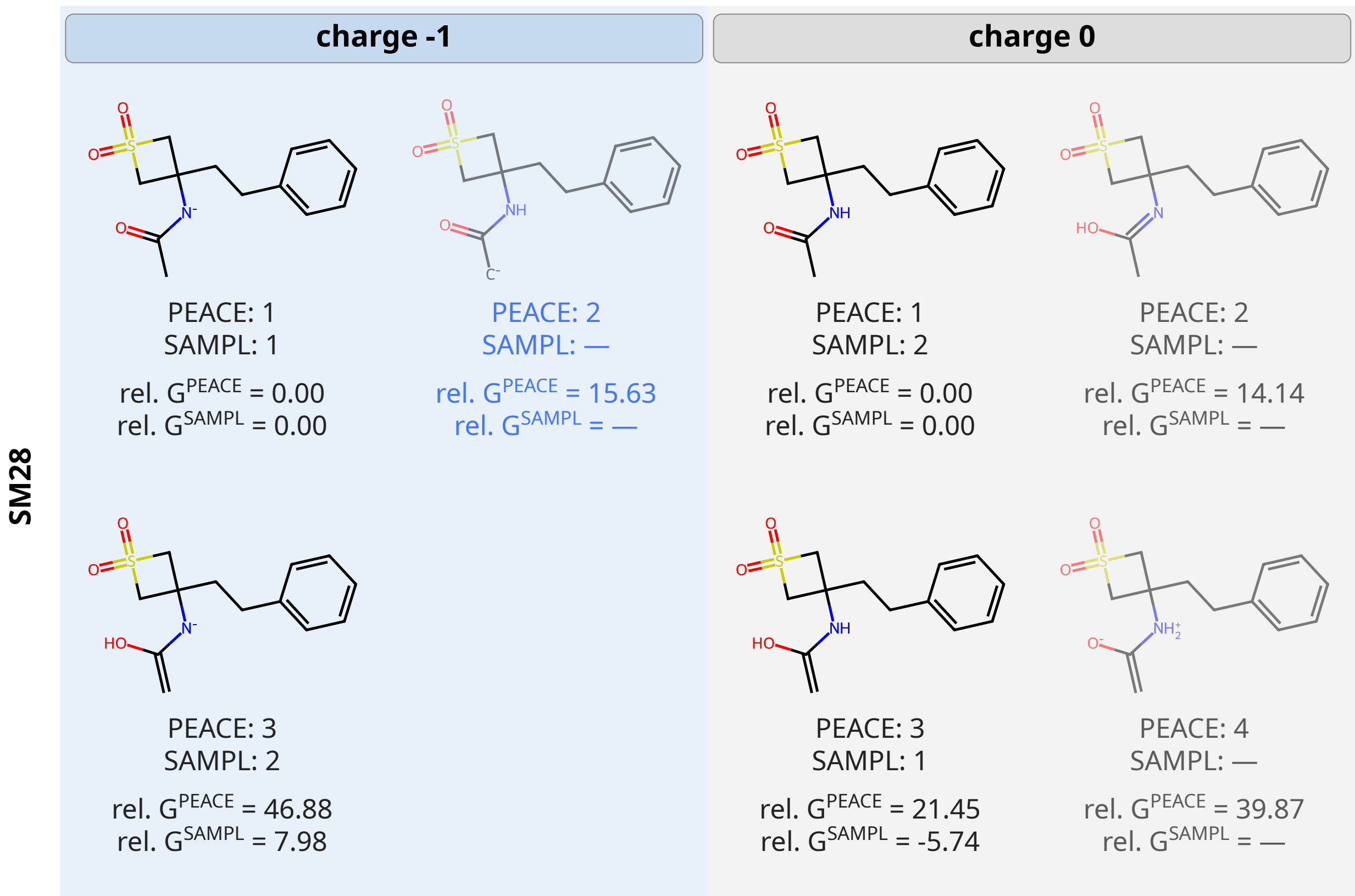

Figure S5: Predicted microstates and energies for species SM28.

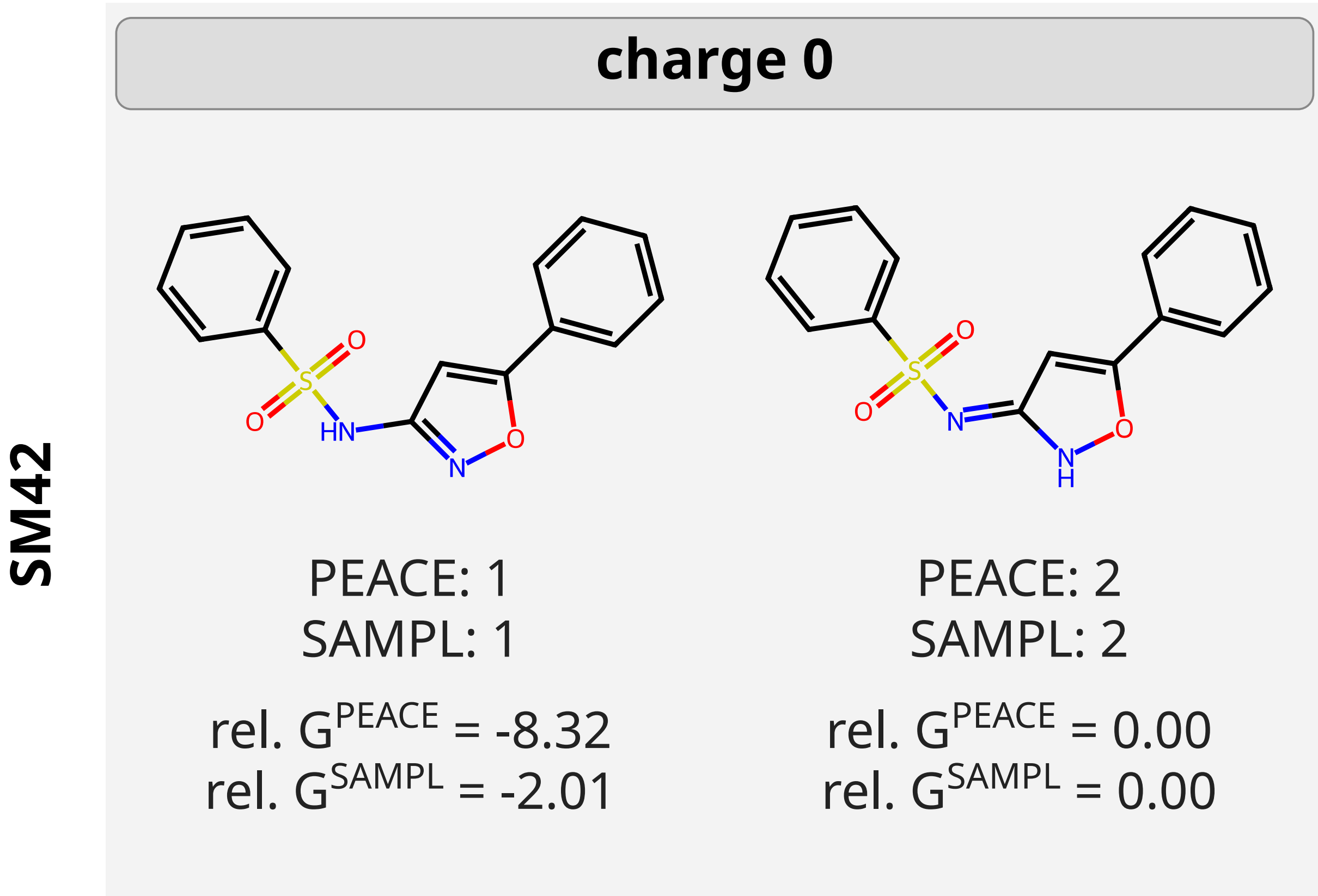

Figure S6: Predicted microstates and energies for species SM42.

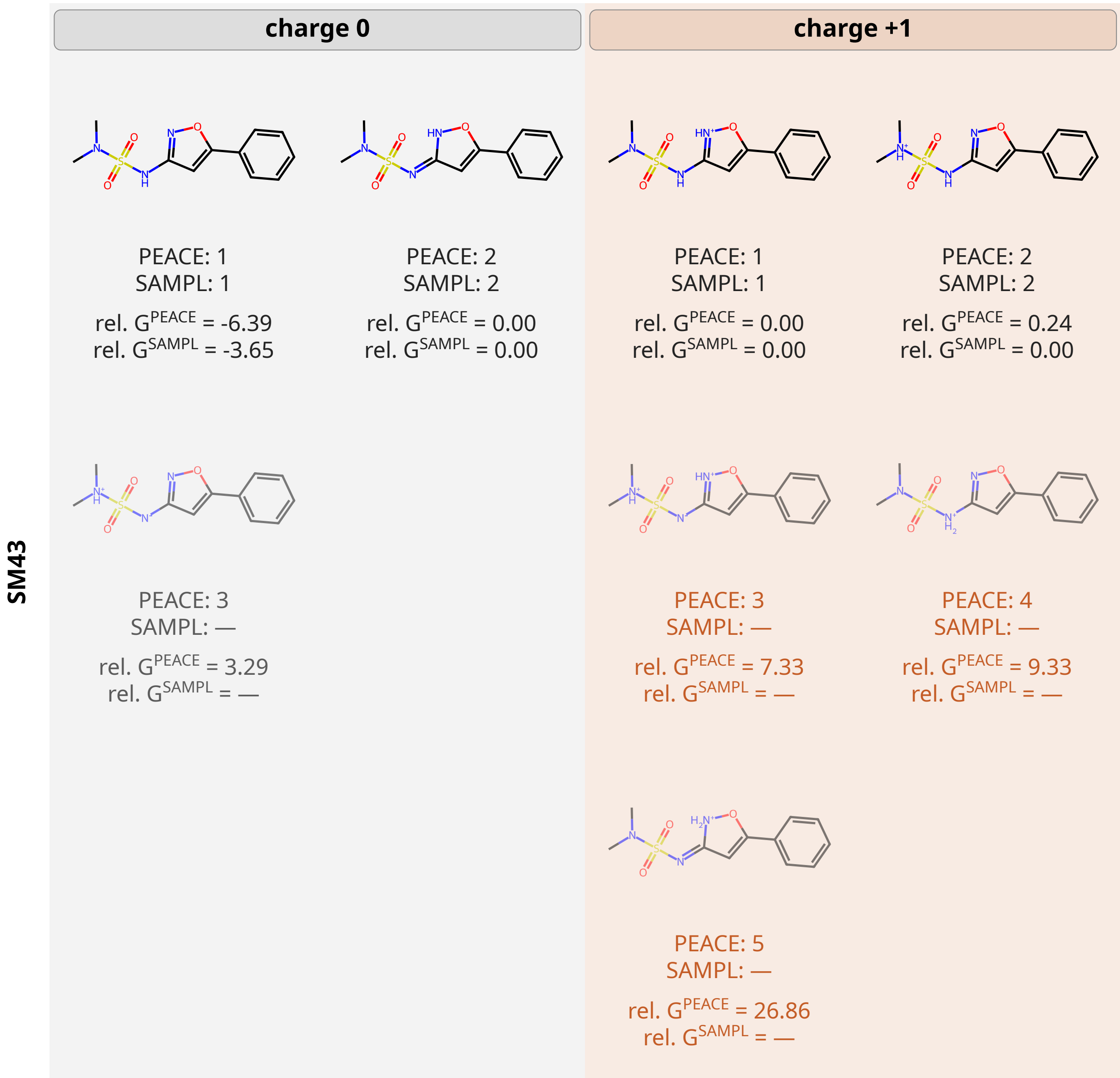

Figure S7: Predicted microstates and energies for species SM43.

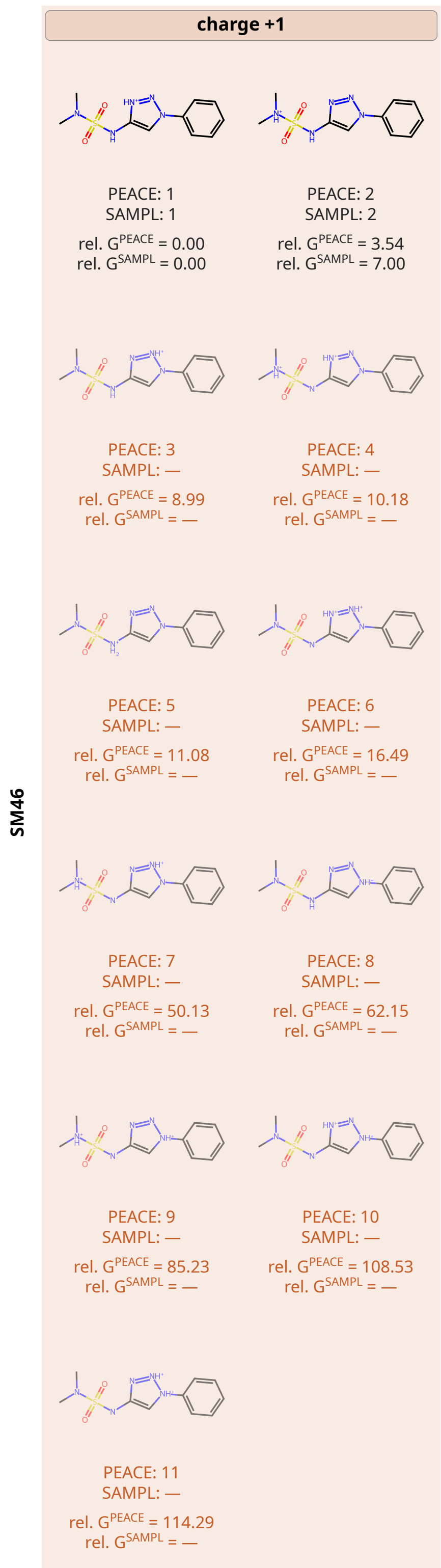

Figure S8: Predicted microstates and energies for species SM46.

1.4 Realistic molecules

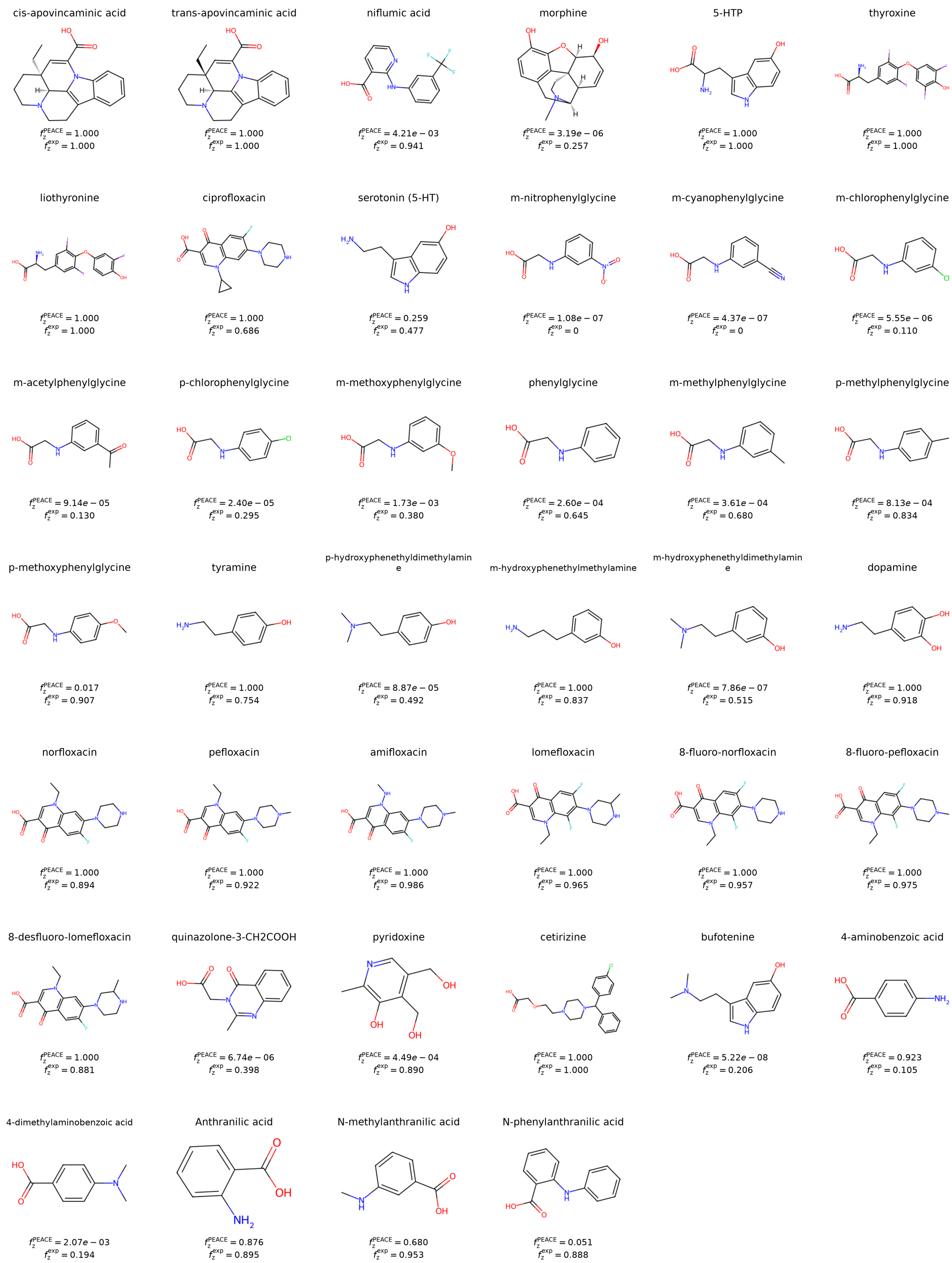

Figure S9: Predicted microstates and populations for the “realistic compounds” set.

2 Benchmarking parameters

Model parameters used in benchmark are shown here:

1. `--screen-threshold` 35.0 kcal/mol (solution free energy under which species are initially pruned for further conformer refinement)

2. `--max-conformers` 10 (maximum number of conformers to consider for conformer refinement)

3. `--embedded-conformers` default $\left(\max[20, \min(3^{n_{\text{rot. bonds}}}, 500)]\right)$

4. `--conformer-energy-threshold` 10.0 kcal/mol (energy window in MMFF94 under which conformers are kept for further refinement)

Higher thresholds (500 kcal/mol for the screen threshold, min 500 embedded conformers, and 40.0 conformer energy threshold) were used for the Gimadiev tautomer set, because only two tautomeric forms are examined at any given time (reducing computational cost), allowing more accurate calculation methods.

3 Corrected Gimadiev values

From the main data compilation, we corrected in total 12 values, 11 of which are from wrong equilibrium (e.g. tabulated value missing a minus sign), and 1 of which is from a seemingly erroneous value. In the 11 wrong-equilibrium cases, the error stems from the original Palm tabulation. For those, we simply added a minus sign to correct the values. In the remaining error, the value in the digital dataset was provided for a zwitterion tautomerization, but matched that of a pyridol-pyridone tautomerism in the Hand book. Therefore, we discarded that value.

From the test set, we corrected 1 equilibrium direction.

The corrected entries, as well as the full and downsampled datasets, are provided in the GitHub repository: https://github.com/jonwzheng/PEACE_experiments/

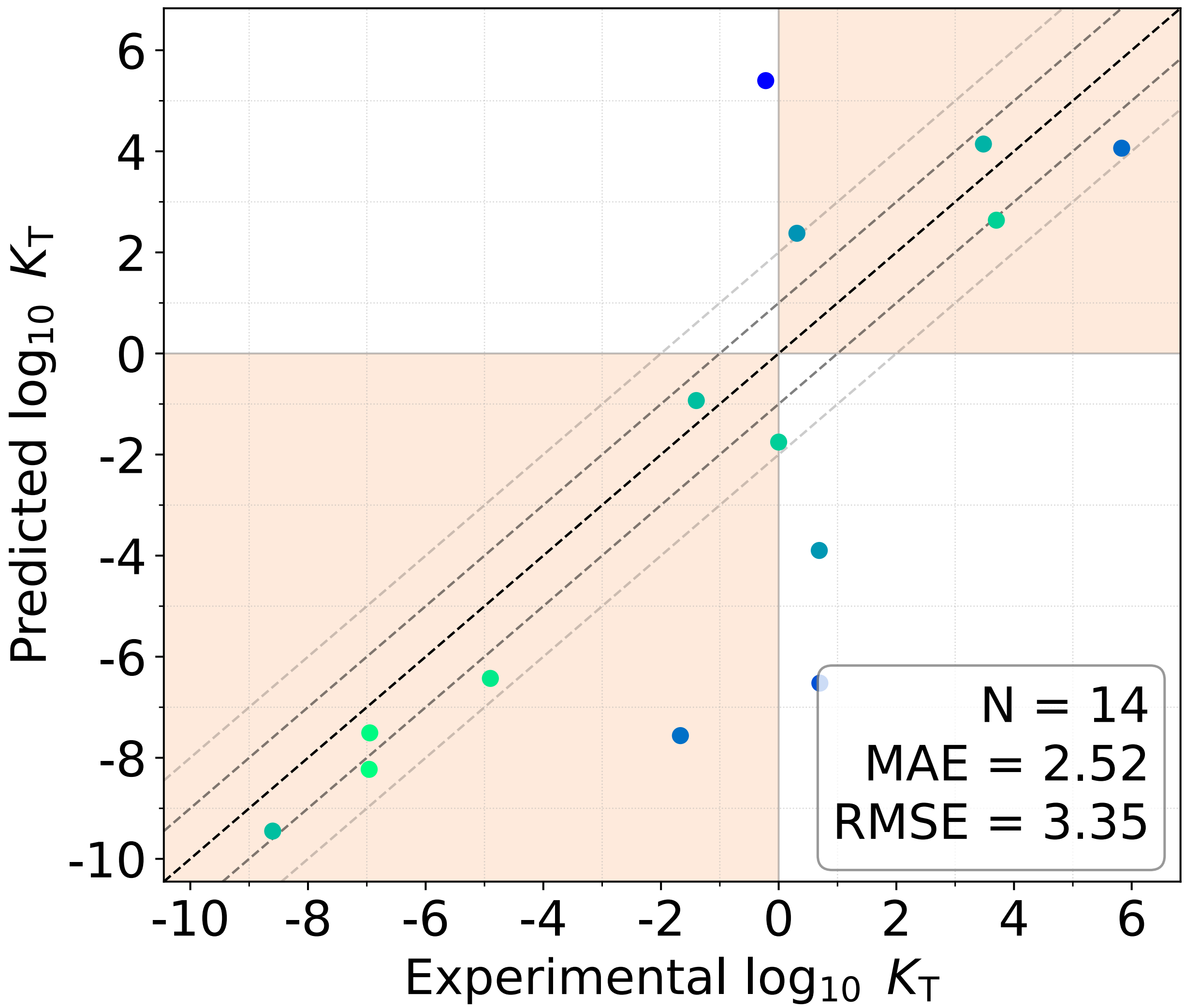

Figure S10: Benchmark of Gimadiev tautomerizations on test set 2, where K_{T} is the tautomerization constant. Orange regions indicate areas where both the experimental and predicted K_{T} agree on the major form. Lighter points indicate higher density of points.
